# Review of Electrochemical Biosensors for Food Safety Detection

**DOI:** 10.3390/bios12110959

**Published:** 2022-11-02

**Authors:** Ke Wang, Xiaogang Lin, Maoxiao Zhang, Yu Li, Chunfeng Luo, Jayne Wu

**Affiliations:** 1 Key Laboratory of Optoelectronic Technology and Systems of Ministry of Education of China, Chongqing University, Chongqing 400044, China; 2 Department of Electrical Engineering and Computer Science, The University of Tennessee, Knoxville, TN 37996, USA

**Keywords:** electrochemistry, biosensor, food safety, high sensitivity, high selectivity

## Abstract

Food safety issues are directly related to people’s quality of life, so there is a need to develop efficient and reliable food contaminants’ detection devices to ensure the safety and quality of food. Electrochemical biosensors have the significant advantages of miniaturization, low cost, high sensitivity, high selectivity, rapid detection, and low detection limits using small amounts of samples, which are expected to enable on-site analysis of food products. In this paper, the latest electrochemical biosensors for the detection of biological contaminants, chemical contaminants, and genetically modified crops are reviewed based on the analytes of interest, electrode materials and modification methods, electrochemical methods, and detection limits. This review shows that electrochemical biosensors are poised to provide miniaturized, specific, selective, fast detection, and high-sensitivity sensor platforms for food safety.

## 1. Introduction

Safe food is a fundamental need for human health. Food safety can be affected by harmful substances such as allergens, pathogens (e.g., parasites, bacteria, viruses, prions, etc.), toxic agents or radioactive substances [1]. To safeguard human health, regulatory agencies such as the United States Food and Drug Administration (USFDA), the European Food Safety Authority (EFSA), and the Chinese Food and Drug Administration (CFDA) have imposed limits on the maximum levels of various contaminants in food. Nevertheless, in 2015, the World Health Organization (WHO) estimated that more than 600 million cases of foodborne diseases and 420,000 deaths are likely to occur each year, due to foodborne diseases caused by 31 foodborne pathogens at the global and subregional levels [2]. In agriculture, pesticides control pests and diseases in crops and ensure crop yield and quality. However, the overuse of pesticides can leave residues on crops that threaten human health through the food chain [3]. In addition, many food additive safety incidents had occurred around the world, such as aquatic products containing malachite green, red-hearted duck eggs dyed with Sudan red, melamine milk powder, industrial gelatin yogurt, etc., causing distrust and fear among people [4]. In order to screen and monitor the safety of food and prevent harm from food contaminants, a sensitive and reliable on-site analysis technology for food contaminants is highly desired.

**Figure 1:**
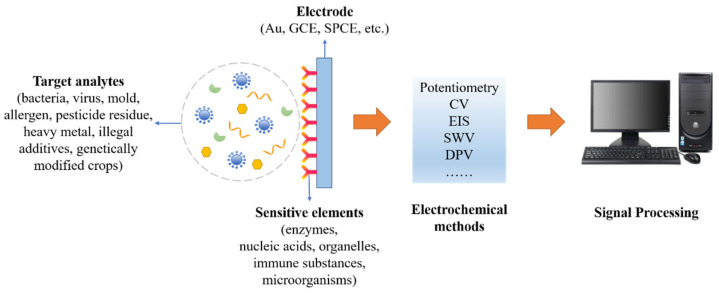
Schematic diagram of an electrochemical biosensor for food contaminants detection.

At present, there are many mature technologies for food safety detection, such as gas chromatography (GC), high-performance liquid chromatography (HPLC), gas chromatography-mass spectrometry (GC-MS), liquid chromatography-mass spectrometry (LCMS), and enzyme-linked immunosorbent assay (ELISA) [5]. However, most of these methods have disadvantages, such as complicated operation, high detection costs, long detection time, and high requirements for the samples tested, which are prone to false positives. To improve this situation, simple, rapid, economical, and portable electrochemical biosensors have attracted much attention. They could not only achieve high specificity and sensitivity, but also enable real-time monitoring in the field. The basic components and principles of an electrochemical biosensor for food contaminants detection are shown in Figure 1. It can convert the biological signal generated by the specific combination of the target analytes and the sensitive elements into an electrical signal, which is detected by the electrochemical methods. Finally, signal processing is performed by a computer to achieve quantitative or qualitative detection of food contaminants.

Common electrochemical methods include potentiometry, cyclic voltammetry (CV), electrochemical impedance spectroscopy (EIS), square wave voltammetry (SWV), and differential pulse voltammetry (DPV). Potentiometry is one of the simplest electrochemical techniques, characterized by a short response time, high selectivity, and extremely low detection limit [6]. Cyclic voltammetry is performed with the applied electrical potential oscillating over a range, while electrochemical impedance spectroscopy is usually performed at a fixed potential over a frequency range [7]. Square wave voltammetry is one of the most advanced and versatile members of the pulse voltammetry technology family, which has high analytical sensitivity and measurement speed [8]. Differential pulse voltammetry is more sensitive than conventional pulse, derivative conventional pulse, and cyclic voltammetry, and is suitable for studying the electrochemical process at the interface of metal-electrolyte solution [9]. Although electrochemical biosensors have not been widely used in food safety detection, their significant advantages deserve further investigation.

**Figure 2:**
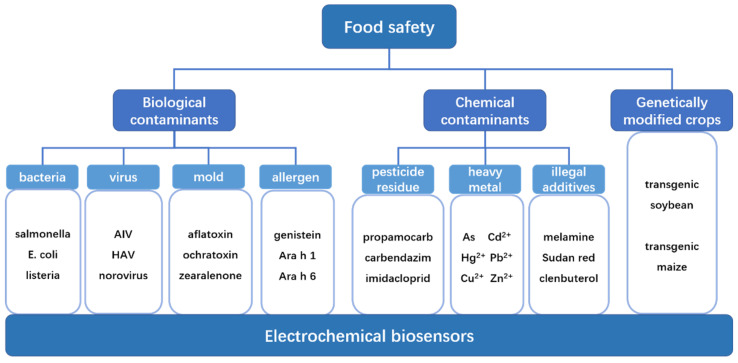
Diagram of the factors affecting food safety.

This review presents various electrochemical biosensors for the detection of biological food contaminants, chemical food contaminants, and genetically modified crops based on the types of analytes of interest. Figure 2 shows the factors that affect food safety. A search was conducted in the Web of Science database using the keywords “electrochemistry”, “food” and the influencing factors in Figure 2, and a total of 96 articles from 2018–2022 were retrieved, from which 31 articles were selected based on the relevance and creativity of the article content. The electrode materials, modification methods, electrochemical methods, linear range, detection limits and detection times of these electrochemical biosensors are discussed in detail. The advantages and disadvantages of these electrochemical biosensors are evaluated based on their selectivity, reproducibility, and sensitivity. The organization is as follows: Section 2, Section 3 and Section 4 analyze the research progress of electrochemical biosensors for the detection of biological food contaminants, chemical food contaminants, and genetically modified crops, respectively. Section 5 discusses the methods to improve the performance of electrochemical biosensors in food safety testing and summarizes the development prospects and future challenges of electrochemical biosensors in the field of food safety testing. This review features a discussion of electrochemical biosensors related to genetic engineering, microfluidics, and molecular imprinting analysis, which can be instructive for the innovation of such sensors.

## 2. Biological Contamination

Biological contaminants include allergens, mycotoxins, pests, and microorganisms [10]. In the process from raw material production to final consumption, food may become contaminated with biological or chemical agents through contact with polluted water, air, soil, and food processing environment [11]. In the following, different electrochemical sensing strategies for the detection of bacteria, viruses and molds are discussed based on recent literature reports.

### 2.1. Bacteria

Bacteria are microorganisms that usually exist in the environment and food matrices, including meat, poultry, fish, eggs, unpasteurized milk, and dairy products [12]. Salmonella, Escherichia coli, and Listeria are among the most monitored bacteria for food safety, which cause foodborne illness in the gastrointestinal tract after consumption. Electrochemical biosensors for the detection of Salmonella, Escherichia coli and Listeria are discussed below (Table 1).

Salmonella is one of the leading causative pathogens responsible for foodborne disease outbreaks. Traditional methods for detecting Salmonella in food are based on culture, including pre-enrichment, selective enrichment, and selective differential plating, and require at least 24 h of preconcentration to increase the number of target bacteria and reach the detection limit of the assay [13]. Biosensing methods with fast response, high sensitivity, and easy use are of great interest for field applications.

**Figure 3:**
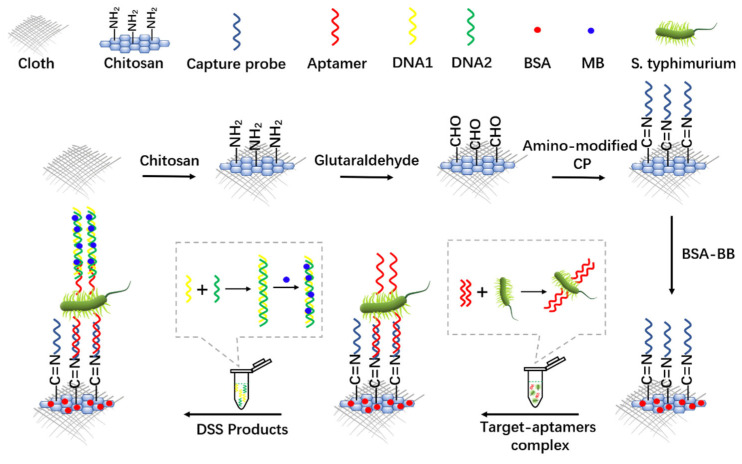
CSEA schematic for direct detection of Salmonella typhimurium (Adapted with permission from Ref. [14]. 2021, Li et al.).

In 2021, Li et al. [14] developed for the first time a novel cloth-based super-sandwich electrochemical aptasensor (CSEA) for the direct detection of Salmonella typhimurium pathogens. Figure 3 is a schematic of CSEA for the direct detection of Salmonella typhimurium. Cloth electrodes and hydrophilic/hydrophobic areas were made using carbon ink and wax-based screen printing as sensing devices. Two specific single-stranded DNA sequences produced a cascading hybridization reaction to form a DNA super sandwich (DSS). Methylene blue (MB) was inserted in its groove to amplify the current signal and thus improve detection sensitivity. The aptamers became bound to Salmonella typhimurium to form a target adaptor complex that could be combined with both the capture probe and DSS to detect Salmonella typhimurium by DPV. In addition, the addition of the aptamer tail sequence made the proposed CSEA universal. In the range of 10^2^ to 10^8^ CFU/mL, the electrochemical signal increased linearly with the logarithmic concentration of Salmonella typhimurium with a detection limit of 16 CFU/mL. In addition, to prove that the aptamer sensor is suitable for the detection of actual samples, milk samples were spiked with 100, 500, and 1000 CFU/mL of Salmonella typhimurium, and the resulting samples were 1:10 diluted with deionized water before testing. CSEA can effectively determine the level of Salmonella typhimurium in milk samples. The prepared CSEA had successfully achieved label-free, nucleic acid-free amplification and cost-effective detection of Salmonella typhimurium. The cloth base used for this sensor is malleable as a flexible substrate, is not easily damaged, and is less costly to mass produce.

In 2022, Yu et al. [15] constructed a proportional electrochemical biosensor based on saltatory rolling circle amplification (SRCA) and dual-signal electrochemical readings. The mercapto-modified β-cyclodextrin (SH-β-CD) was fixed to the surface of the glassy carbon electrode (GCE) by binding to gold nanoparticles (AuNPs) to form Au-S bonds, forming SH-β-CD/AuNPs/GCE. AuNPs not only have good electrical conductivity, but also increase the specific surface area. After the SRCA reaction with primers, a large number of amplification products containing ferrocene (Fc) were obtained within 1 h of double-stranded DNA (dsDNA), which can bind to SH-β-CD through the interaction between the subject and the object. In addition, MB was embedded in the phosphate backbone of dsDNA due to the electrostatic attraction between MB and DNA. SWV was used for electrochemical monitoring; the peak current of the blank group Fc was higher than MB, and the peak current of MB was significantly greater than Fc when Salmonella typhi is present. This ratio metric electrochemical biosensor had a linear detection range of 30 fg/μL to 30 ng/μL with a detection limit of 15.8 fg/μL. By testing food samples, the proportional electrochemical biosensor agrees with RT-qPCR results and the test time is shorter. Therefore, this sensor can be used as an alternative to RT-qPCR.

Foodborne diseases caused by Escherichia coli (*E. coli*) are causing morbidity and mortality worldwide, threatening human health [16]. Contamination by this pathogen plays an important role in the food industry, environment, and health sectors, making *E. coli* detection crucial. However, current analysis procedures require at least 18 h from sample collection to results [17]. Therefore, it is necessary to develop an accurate, sensitive, rapid, and cost-effective method for *E. coli* detection.

**Figure 4:**
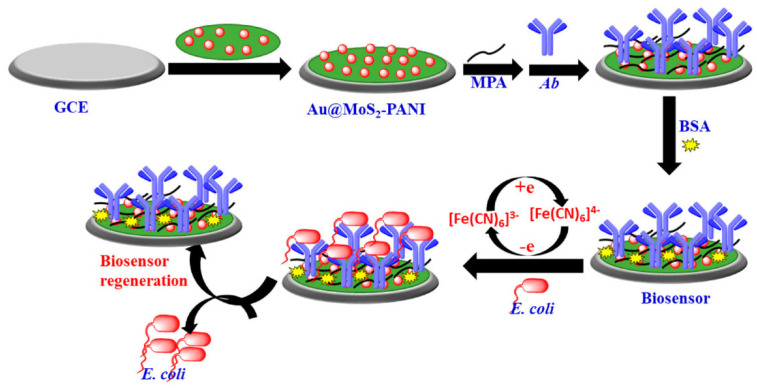
Schematic diagram of electrochemical biosensor for *E. coli* (Reprinted with permission from Ref. [18]. 2021, Raj et al.).

Raj et al. [18] used Au@MoS_2_-polyaniline (PANI) nanocomposites to develop a simple, label-free, and highly sensitive immunosensor based on electrochemical detection for the detection of *E. coli*. PANI and AuNPs can increase the conductivity and surface area of MoS_2_ and improve the conductivity of GCE. The sensing strategy of the immune sensor is shown in Figure 4. Self-assembled thiol propionate monolayers were introduced on the surface of the AuNP to covalently immobilize antibodies and prevent nonspecific adsorption of the target pathogen on the electrode surface. CV and DPV were used to confirm the successful preparation of the biosensor on the surface of GCE, and EIS was used to characterize the electrochemical performance of the modified electrodes. CV and DPV experiments were performed in 5 mm [Fe(CN)_6_]^3-/4-^ containing 0.1M KCl solution. The peak value of DPV current in [Fe(CN)_6_]^3-/4-^ decreased with increasing *E. coli* concentration on the electrode surface due to the formation of antibody-antigen complexes between *E. coli* and the antibodies of the biosensor, resulting in a spatial barrier to electrical current from the solution to the electrode surface. The biosensor achieved simple and sensitive detection of *E. coli* as low as 10 CFU/mL within 30 min, with a linear detection range of 10–107 CFU/mL. The sensor is capable of detecting *E. coli* in urine samples and the electrodes may be regenerated. However, it has high sample requirements and cannot accurately detect *E. coli* in clinical samples with a complex composition such as serum, sputum, and whole blood.

**Figure 5:**
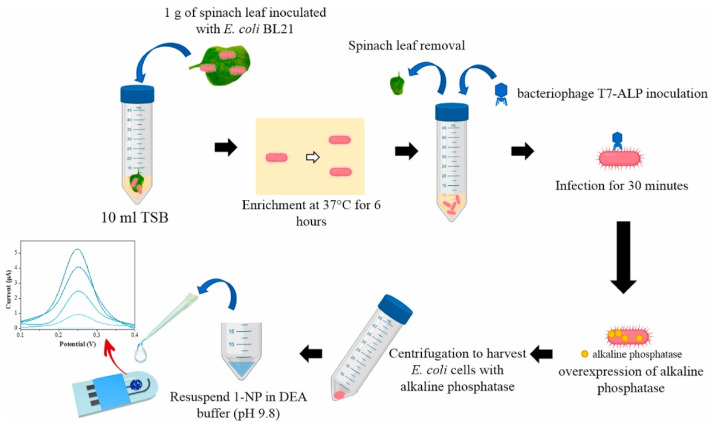
Schematic diagram of a phage electrochemical biosensor for the detection of *E. coli* on spinach leaves (Reprinted with permission from Ref. [19]. 2022, El-Moghazy et al.).

El-Moghazy et al. [19] developed a genetically engineered phage T7-based electrochemical biosensor for the rapid detection of *E. coli* in fresh produce. The sensing platform inserted the gene encoding alkaline phosphatase (ALP) into the T7 phage genome to form a genetically engineered phage, which was used as a biorecognition element, and targeted cleavage of the target bacteria by the phage can trigger overexpression of ALP. As shown in Figure 5, 1 g of spinach leaves purchased from a local supermarket were weighed, placed in a sterile Petri dish, and then inoculated with different concentrations of *E. coli*. The overexpression of ALP was tracked electrochemically using a single-walled carbon nanotube-modified screen-printed electrode (SWCNT-SPE), and electrochemical measurements were performed by DPV. The current signal increased with increasing *E. coli* concentration, and the peak current was linear in the range of 1–10^4^ CFU/mL versus the logarithm of the bacterial concentration. The detection limit was 1 CFU/mL. The electrochemical sensor was capable of rapid and accurate quantitative detection of pathogenic *E. coli* on spinach leaves within 1 h after pre-enrichment. In addition, this biosensor exhibited high specificity for *E. coli* in the presence of other common food bacterial contaminants and reduced analysis time through the coupling between specially designed phage and electrochemical methods. Compared to the study by Raj et al. [18], the method reduces complexity, does not require expensive materials, and has a lower detection limit, which can be extended to highly sensitive and selective detection of different bacterial contaminants in food samples, but with a small linear range and longer detection time.

Listeria monocytogenes (LM) can cause Listeriosis, a serious food-borne infection that is mainly seen in immunocompromised patients. It also leads to maternal and neonatal infections, mainly manifesting as sepsis and neuropathy [20]. In recent years, electrochemiluminescence (ECL) [21], cell culture [22], ELISA, and oligonucleotide-based sensors [23] have been successfully established for the detection of Listeria monocytogenes in the food industry. Nonetheless, the detection sensitivity of the first three methods is not sufficient to determine trace levels of monocytobacter, thus limiting their utility.

**Figure 6:**
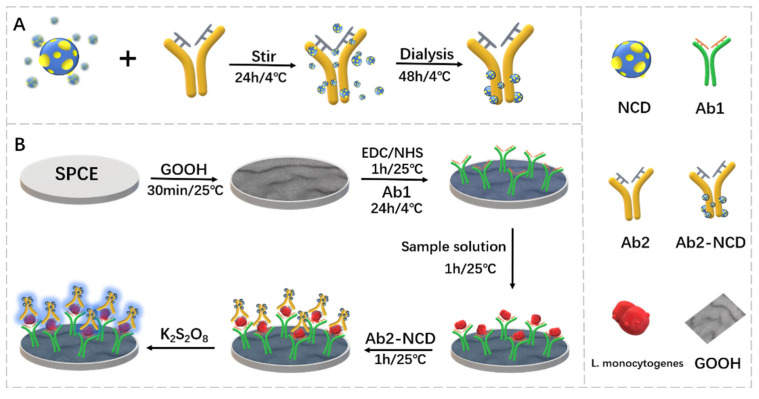
Schematic of (**A**) NCD conjugated with secondary antibody and (**B**) the developed ECL sensor for monocytic bacteria (Adapted with permission from Ref. [24]. 2021, Jampasa et al.).

To overcome these limitations, Jampasa et al. [24] designed an ultra-sensitive electrochemiluminescence sensor based on nitrogen-modified carbon dots (NCDs) for the determination of LM using screen-printed carbon electrodes (SPCE), combining the advantages of earlier developed ECL methods. The preparation and detection processes of the sensor are shown in Figure 6. Carbon dots (CDs) were synthesized using citric acid as the carbon source and ethylenediamine (a molecule containing nitrogen atoms) as the nitrogen source. Approximately 4 nm NCD with uniform size distribution can be prepared by a one-step green microwave-assisted method. Carboxyl graphene (GOOH) was used as an electrode modifier to directly introduce the assigned functional groups and to increase the conductivity of the proposed platform. The ECL sensor was constructed by modifying SPCE with GOOH, activating the surface of the GOOH-modified SPCE with EDC/NHS at room temperature, and then immobilizing the trapping antibody (Ab1) on the GOOH-modified SPCE. The addition of the immune complex Ab2-NCD resulted in a significant increase in the ECL signaling response in the presence of K_2_S_2_O_8_. ECL signal gradually monotonically increased with the further increase in monocytosis concentration. This modified ECL sensor has a LOD of 1.0 × 10^−1^ CFU/mL, a linear range of 2 to 1.0 × 10^6^ CFU/mL, and a sensitivity of 1.0 × 10^−1^ CFU mL^−1^. The accuracy and precision of the sensor were confirmed by measuring LM in milk, sausage and ham and comparing the results with the standard SO 11290-2:2017 method (horizontal method for Listeria monocytogenes). Nitrogen doping is a key step in improving the signal of this sensor, making it competitive with other related LM sensing platforms in terms of sensitivity, reproducibility and operational cost.

**Figure 7:**
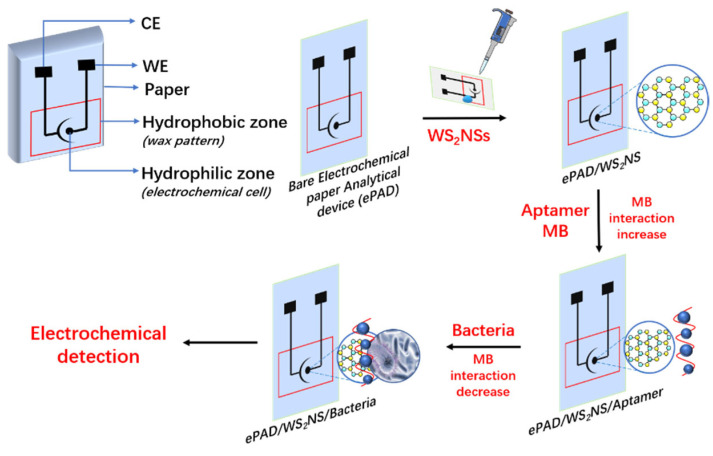
Schematic of a screen-printed paper-based aptamer sensor for the detection of Listeria monocytogenes (Adapted with permission from Ref. [25]. 2022, Mishra et al.).

Mishra et al. [25] reported a novel aptamer sensor based on an electrochemical paper-based analytical device (ePAD). As shown in Figure 7, the device used tungsten disulfide (WS_2_) and aptamer modifications to detect Listeria monocytogenes on a screen-printed paper electrode. EIS was used to analyze ePADs obtained after modification by WS_2_ nanostructures (WS_2_N_S_), aptamer ssDNA, and bacteria. EIS is not only for characterizing the step-by-step assembly of ePADs, but also to evaluate the sensing performance of Listeria monocytogenes. The experimental results show that the resistance value increased with the increase in aptamer concentration, which was attributed to the DNA aptamer depositing an insulating layer on the surface of ePAD. In the linear range of 10^1^–10^8^ CFU/mL, the detection limit and quantitative limit of the nucleic acid aptamer sensor are 10 and 4.5 CFU/mL. The sensor successfully detected LM in dairy samples. This aptamer sensor uses a paper-based platform as a substrate, reducing the manufacturing cost of the sensor and the volume of analyte required, facilitating mass production and application.

**Table 1:** Electrochemical biosensors for bacterial detection.

Analyte	Electrode	Electrochemical Method	Linearity Range	LOD	Assay Time	Ref.
Salmonella	SPCIE	DPV	10^2^–10^8^ CFU/mL	16 CFU/mL	—	[14]
	GCE	SWV	30 fg/μL–30 ng/μL	15.8 fg/μL	—	[15]
*E. coli*	GCE	DPV	10–10^7^ CFU/mL	10 CFU/mL	30 min	[18]
	SPE	DPV	1–10^4^ CFU/mL	1 CFU/mL	1 h	[19]
Listeria	SPCE	CV	2–1.0 × 10^6^ CFU/mL	0.1 CFU/mL	—	[24]
	SPPE	EIS	10^1^–10^8^ CFU/mL	10 CFU/mL	—	[25]

### 2.2. Virus

Viruses are a common cause of foodborne disease outbreaks. Viral diseases have a low mortality rate but can be transmitted to humans through food due to improper food handling [26]. The rapid replication and high transmissibility of the virus can have serious consequences not only for individuals but also for the health of communities, and even cause serious economic impacts. Therefore, a quick, accurate, and low-cost test diagnostic tool for a large number of people is very important. In the following, electrochemical biosensors for common avian influenza virus, hepatitis A virus, and norovirus are discussed (Table 2).

**Figure 8:**
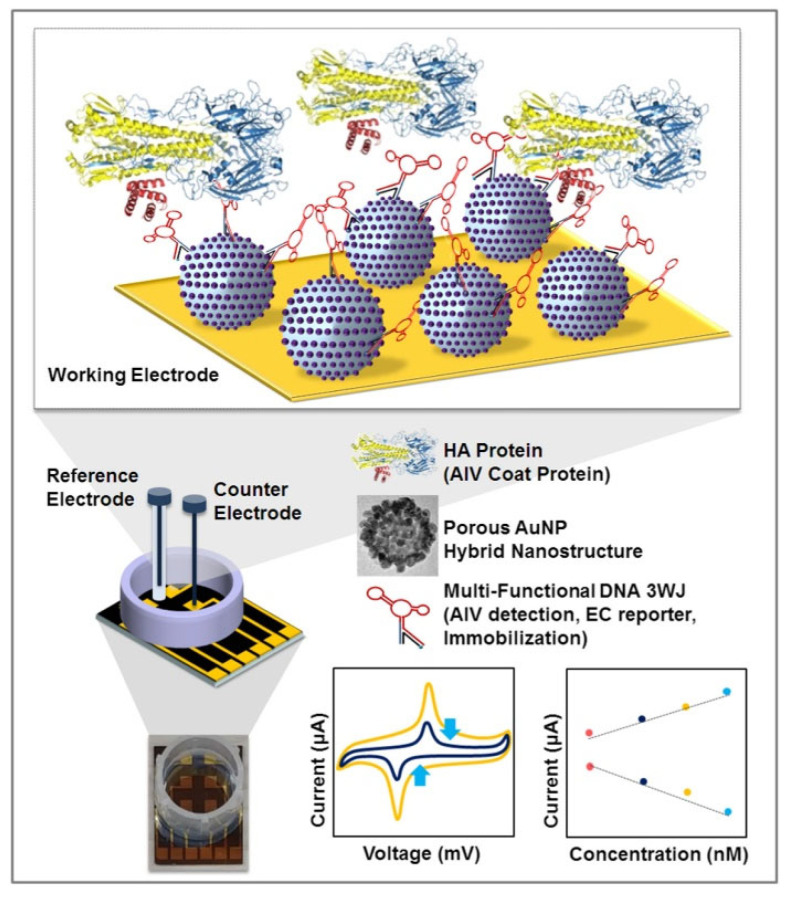
Schematic diagram of a biosensor for detection of avian influenza virus (Reprinted with permission from Ref. [28]. 2019, Lee et al.).

Avian influenza virus (AIV) is one of the pathogens that endanger human health. Avian influenza viruses can also affect the safety of food supply and cause significant economic losses [27]. Therefore, an accurate, sensitive, and fast detection method is the key to decision-making. Lee et al. [28] used electrochemical technology to prepare a label-free AIV H5N1 biosensor composed of multifunctional DNA structure on electrodes made of porous gold nanoparticles (pAuNPs), and its structure and detection principle are shown in Figure 8. DNA 3 way-junction (3WJ) was introduced as a multifunctional bioprobe, and each fragment was assembled into DNA 3WJ for AIV detection, and the assembly structure was confirmed by native magnesium trisborate polyacrylamide gel electrophoresis (TBM-PAGE). In order to improve the sensitivity of electrochemical signals, pAuNPs were synthesized, and DNA 3WJ was modified on PaUnPS-modified gold electrodes by a layer-by-layer (LbL) assembly method. The surface morphology of pAuNPs-modified gold electrodes was studied by FE-SEM and AFM. The binding of HA protein to DNA 3WJ modified electrode was confirmed by CV. The manufactured biosensor was observed to have a linear range of 1 pM-100 nM in HEPES buffer, a LOD of 9.43 pM, and 1 μM HA protein could be detected in diluted chicken serum. Each DNA fragment of the sensor has a specific function. These nucleic acid fragments are well assembled and there is no loss of function. Compared to other reports, the sensor does not require additional labeling and signal amplification processes and can be applied for multi-target detection.

**Figure 9:**
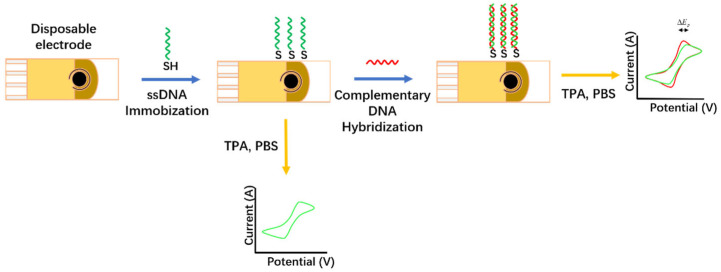
Disposable DNA biosensor constructed by thiol gold coupling of thiolated single-stranded DNA probes (Adapted with permission from Ref. [30]. 2018, Manzano et al.).

Hepatitis A virus (HAV) can be transmitted through the fecal-oral route, which is mainly related to the ingestion of food or water contaminated with infected feces and is a common cause of clinical hepatitis and acute liver failure [29]. Manzano et al. [30] developed an electrochemical method based on DNA pairing to detect the hepatitis A virus. Its sensing strategy is shown in Figure 9. A single-stranded DNA probe (capture probe) was designed for the hepatitis A virus and the DNA of samples containing the virus was detected by nested reverse transcriptase polymerase chain reaction (RNT-PCR). To develop electrochemical devices, disposable gold electrodes were functionalized using specific capture probes and tested on complementary single-stranded DNA and HAV cDNA. CV was used to monitor the oxidation peak potential of the indicator tripropylamine, and the DNA pairing on the electrode was measured. To prevent non-specific binding, the gold surface was treated with 3% BSA before assay. High-resolution atomic force microscopy (AFM) confirmed the efficiency of electrode functionalization and on-electrode pairing. The linear range of HAV detection was 10 fg/μL–10 pg/μL, and the detection limit was 1.08 fg/µL. This electrochemical analysis method is less time-consuming than conventional PCR analysis. The sensor has shown comparable sensitivity to nRT-PCR assays, in addition to its great potential to reduce costs and time in hepatitis A virus detection.

**Figure 10:**
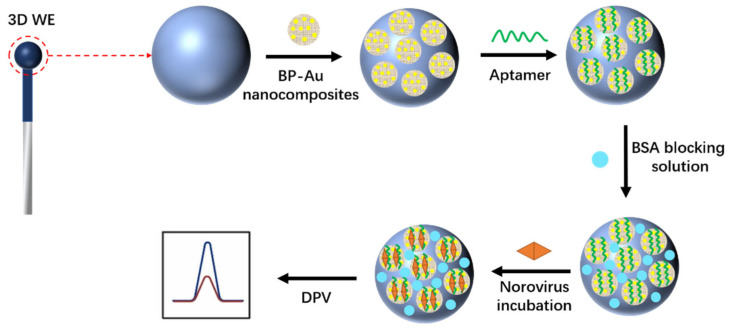
Schematic of a 3D electrochemical aptamer sensor for the detection of norovirus (Adapted with permission from Ref. [32]. 2022, Jiang et al.).

Norovirus is one of the causes of a high degree of gastrointestinal infection, mainly from contaminated water or contaminated food [31]. Focusing on promising alternatives for more sensitive and accurate detection of norovirus, Jiang et al. [32] constructed a 3D electrochemical aptamer sensor for norovirus-sensitive detection. As shown in Figure 10, it is characterized by the modification of movable spherical working electrodes (WE) with gold phosphate nanocomplexes (BP-AuNCs). The BP-AuNCs were prepared by restoring phosphorene nanosheets (BPNSs) in situ on chloroauric acid (HAuCl_4_). The removable spherical WE was made by hand applying carbon ink to the ball head and then baking it in the oven at 50 °C, a design that helps increase surface area, simplify sampling, and avoid cross-contamination. Mercaptan-modified aptamers can easily bind to the surface of BP-AuNCs by covalent bonding without altering the structural or functional properties of aptamers. This sensing strategy is based on a specific binding between norovirus and aptamers, and then the resulting complex can cause diffusion barriers on the WE, altering the electrochemical signal. DPV was used to characterize the electrochemistry of 3D aptamer sensors with a detection limit of 0.28 ng/mL and a linear range of 1 ng/mL to 10 μg/mL. The proposed 3D electrochemical aptamer sensor had been successfully applied to the detection of norovirus in oyster samples with an average detection time of 35 min. The spherical working electrode design effectively reduces the size of the sensor and facilitates the miniaturization of the sensor. The sensor provides a simple, low-cost strategy for sensitive and selective detection of norovirus.

**Table 2:** Electrochemical biosensors for virus detection.

Analyte	Electrode	Electrochemical Method	Linearity Range	LOD	Assay Time	Ref.
AIV	Au	CV	1 pM–100 nM	9.43 pM	—	[28]
HAV	Au	CV	10 fg/μL–10 pg/μL	1.08 fg/µL	—	[30]
Norovirus	CIE	DPV	1 ng/mL–10 μg/mL	0.28 ng/mL	35 min	[32]

### 2.3. Mold

Mycotoxin contamination of food and feed is considered one of the most serious food safety problems in the world because these fungal metabolites may be teratogenic, mutagenic, carcinogenic, and immunosuppressive, and may seriously damage animal and human health. Early common mycotoxin detection methods mainly include high-performance liquid chromatography, thin layer chromatography, mass spectrometry, gas chromatography, and ELISA [33]. These methods provide accurate and reliable detection of mycotoxin, but these methods have some problems, such as complex sample preparation and long detection time. Electrochemical biosensors have attracted much attention in mycotoxin analysis because of their rapid, sensitive, specific, and portable advantages. The following is a description of several electrochemical biosensing strategies used to detect aflatoxin, ochratoxin, and zearalenone (Table 3).

**Figure 11:**
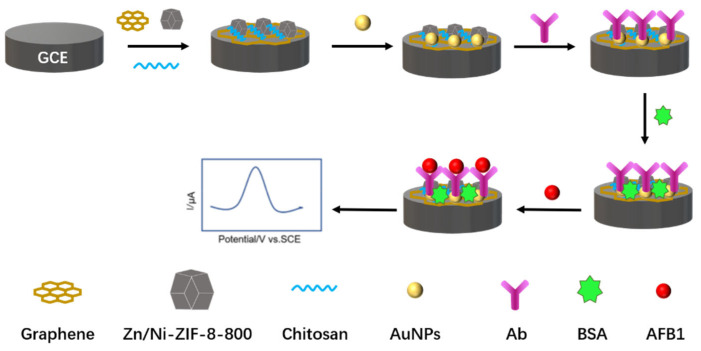
Schematic of an electrochemical immunosensor for the detection of aflatoxin B1 (Adapted with permission from Ref. [35]. 2022, Wang et al.).

Aflatoxin has a carcinogenic effect and is one of the most important factors affecting the safety of the food industry [34]. To meet the detection requirements, Wang et al. [35] developed a simple, efficient, and sensitive electrochemical immunosensor for the detection of aflatoxin B1 (AFB1) in peanut oil. Figure 11 is a schematic of an electrochemical immunosensor for the detection of aflatoxin B1. Bare GCEs were modified with graphene, bimetallic organic framework materials (Zn/Ni-ZIF-8-800), chitosan and AuNPs. Electrochemical immunosensors were characterized by CV and the changes in electrochemical signals after antibody and AFB1 binding were studied in detail. The results show that the electrochemical reaction of AFB1 on this electrochemical immunosensor is a diffusion control process. The signal response of AFB1 at different concentrations was measured with DPV, and the DPV response signal gradually decreased as the AFB1 concentration increased. Electrochemical immunosensors have a linear range of 0.18–100 ng/mL and a detection limit of 0.18 ng/mL. The prepared sensors provide the basis for simple, fast and sensitive detection of AFB1 in peanut oil. Although modification of the electrodes with graphene, Zn/Ni-ZIF-8-800, chitosan and AuNPs can improve the electrode surface conductivity, the material preparation process is complex and requires specific reagents, equipment, and methods, which is operationally difficult for non-specialists.

Mazaafrianto et al. [36] developed an electrochemical sensor for the quantitative detection of ochratoxin A(OTA) by using an aptamer with two dithiol groups that exhibited higher stability on gold electrodes than A single thiol-based aptamer. The sensor is also based on a signal-on scheme that generates a signal current through structural switching of aptamers upon interaction with the OTA. To simplify the fabrication of the sensor, the non-covalent interaction of methylene blue with the aptamer was also used as an electrochemical indicator. In addition, the sensor performance was characterized, including the dissociation constants of the aptamer-OTA complex. The sensor is highly reproducible and sensitive enough to detect the minimum amount of OTA required for the analysis of actual food samples, with a detection limit of 113 pM. The aptamer modification scheme of the sensor has been successfully applied to microprocessed electrodes. By varying the appropriate aptamer according to the target analyte, the sensor is able to provide a simple, portable and versatile platform for in situ detection.

Zearalenone (ZEA) is a mycotoxin produced by Fusarium fungi, which is highly toxic to animal and human health [37]. Radi et al. [38] used CV, DPV, and EIS to study the electrochemical behavior of zearalenone on a single-wall carbon nanotube screen printing electrode (SWCNT-SPCE) and observed a single irreversible oxidation peak. Due to the adsorption of ZEA on the electrode surface, the peak DPV current on SWCNT-SPCE was significantly enhanced. Maize GA was determined by differential pulse adsorption vapor voltammetry (DPASV). Under optimized conditions, the peak anode current of ZEA varies linearly with ZEA concentration in the range of 2.5 × 10^−8^–1.0 × 10^−6^ M, and the detection limit is 5.0 × 10^−9^ M. The results showed that the method could be used for the quantitative analysis of zearalenone in cornflakes. It is worth mentioning that the electrode surface of the sensor is not contaminated by oxidation products and the electrodes can be reused, reducing the cost of the sensor.

**Table 3:** Electrochemical biosensors for mold detection.

Analyte	Electrode	Electrochemical Method	Linearity Range	LOD	Assay Time	Ref.
Aflatoxin	GCE	DPV	0.18–100 ng/mL	0.18 ng/mL	—	[35]
Ochratoxin	Au	DPV	0.25–750 nM	113 pM	—	[36]
Zearalenone	SWCNT-SPCE	DPASV	2.5 × 10^−8^–1.0 × 10^−6^ M	5.0 × 10^−9^ M	—	[38]

### 2.4. Allergen

Components of food that can cause abnormal reactions in the body’s immune system are known as allergens. National and international agencies are enacting laws, regulations, and food labeling standards to avoid allergies in people by labeling allergen components on foods [39]. Rapid on-site detection of allergic components in food can safeguard the health and quality of life of food allergy patients [40]. Currently, electrochemical biosensors have become an important tool for the rapid detection of food allergens, and the electrochemical biosensors used for the detection of soybean and peanut allergens are discussed below (Table 4).

Sundhoro et al. [41] applied MIPs for the first time to achieve the detection of the soybean allergenicity marker genistein in complex foods. The electrode coated with MIPs showed high sensitivity (lower limit of detection of 100 ppb) and successfully distinguished genistein from several structurally similar isoflavone and flavonoid molecules. The sensor’s results for soybean allergens were comparable to or better than the performance of other commercially available portable allergen detection devices, including lateral flow devices (LFDs) and ELISA. Although the sensor’s performance has not yet exceeded the highest sensitivity for soybean allergens obtained using complex, expensive and labor-intensive methods such as mass spectrometry and PCR, its high selectivity, short detection time, and low-cost offer significant advantages over leading detection technologies.

**Figure 12:**
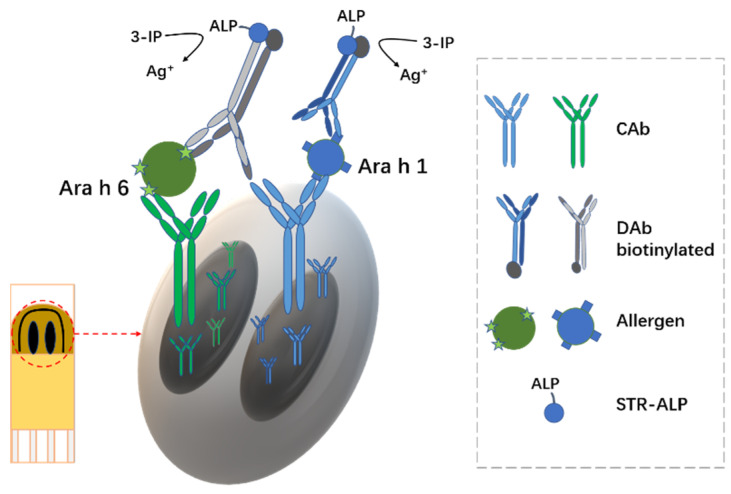
Schematic diagram of the immune sensor configuration and structure (Adapted with permission from Ref. [42]. 2021, Freitas et al.).

Freitas et al. [42] developed an electrochemical dual immunosensor for the simultaneous analysis of two major peanut allergens, Ara h 1 and Ara h 6. As shown in Figure 12, Sandwich immunoassays were performed using monoclonal antibodies on a double-working screen-printed carbon electrode. The assay time was 2 h 20 min and the actual operating time was 30 min. The limit of detection for Ara h 1 was 5.2 ng/mL. The limit of detection for Ara h 6 was 0.017 ng/mL. The dual immunosensor has been successfully applied to the analysis of several food products and was able to quantify peanuts down to 0.05%. The accuracy of the results was confirmed by recovery studies and comparison with ELISA. The sensor is equally suitable for complex food matrices.

## 3. Chemical Contamination

Chemical contamination in food has a wide range of sources, variety and complex compositions. Most foods are inevitably contaminated by pesticides, heavy metals, food additives and other common chemicals [43]. Excessive intake of these substances can have negative effects on human health. In the following, different biosensing strategies for the detection of pesticide residues, heavy metals, and illegal additives are discussed based on existing electrochemical biosensors for food safety detection.

### 3.1. Pesticide Residue

Pesticides can prevent crop pests and diseases, but they accumulate in vegetables, fruits, and meat throughout the food chain [44]. Consumption of food with excessive pesticide residues will endanger human health. At present, the most commonly used pesticide detection methods include GC, GC-MS, liquid chromatography (LC), and LCMS. These methods have high sensitivity, selectivity, and accuracy with the potential for multi-component analysis. However, they are time-consuming and expensive, often requiring sample preparation [45]. In recent years, electrochemical-based biosensors have been investigated to detect pesticide residues in order to overcome the limitations of traditional methods (Table 5).

Nevin et al. [46] used a green ultrasound-microwave assisted method to prepare reduced-GO (rGO) and studied rGO-based non-enzymatic electrochemical sensors for the detection of synthetic fungicides used as propamocarb (PM) pesticides. To test whether the rGO-based non-enzymatic electrochemical sensor detected PM residues in real vegetables, the sensor was tested on cucumber samples with 5 different concentrations of PM pesticides. The sensor was able to detect PM pesticide on actual cucumber samples with high sensitivity within a cycle time of 1 min, with a detection limit as low as 0.6 μm, a sensitivity of 101.1 μA μm^−1^cm^−2^, and a linear range of 1 to 5 μm. The results show that the modification of rGO on the sensor surface can accelerate the response of the sensor. The most outstanding advantage of the sensor is that it takes only 1 min to complete the detection, and can be used in portable detection kits for rapid on-site detection of PM-type pesticides in food.

**Figure 13:**
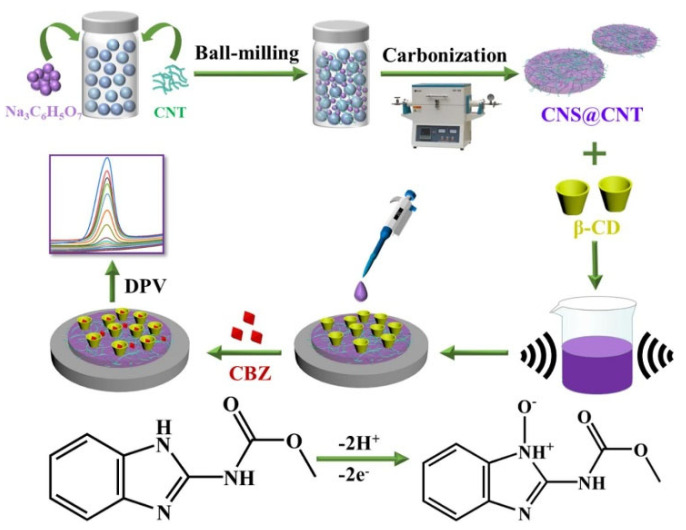
Fabrication schematic of β-CD/CNS@CNT/GCE sensor (Reprinted with permission from Ref. [48]. 2022, Liu et al.).

Carbendazim (CBZ) is one of the most commonly used benzimidazole fungicides. It is widely used to promote food production and its residues pose a great threat to human health and the environment [47]. Liu et al. [48] prepared a novel electrochemical sensor for CBZ determination based on β-cyclodextrin (β-CD) functionalized carbon nanosheets @ carbon nanotubes (CNS@CNT). Its sensing strategy is shown in Figure 13. CNS@CNT combines the large surface area of CNS with the excellent conductivity of CNT to significantly improve the electrocatalytic performance of the sensor. In addition, β-CD is well capable of host GEST supramolecular recognition, which can improve the selective recognition and enrichment ability of CBZ. The β-Cd/CNS@CNT/GCE sensor has a lower detection limit of 9.4 nM in the linear CBZ concentration range from 0.03 to 30 μm. The fabricated sensor has good stability, high sensitivity (30.86 Aμm^−1^cm^−2^), and reliable reproducibility (RSD = 3.6%). In particular, β-CD/CNS@CNT/GCE sensor can be used for the detection of CBZ in apple juice. Compared with the detection performance of other CBZ sensors, the β-CD/CNS@CNT/GCE sensor has a relatively low LOD, good long-term stability, anti-interference, and selectivity.

Imidacloprid (IM) has been widely used in crop pest control and disease resistance, and the high sensitivity detection of imidacloprid concentration has put forward strong requirements in the field of food safety [49]. Tang et al. [50] reported a sensitive ECL sensor based on upconverting nanoparticle functional zeolite imidazolate framework (UCNPs@ZIF-8) nanocomposites combined with molecularly imprinted polymers (MIPs), which successfully realized the quantitative detection of IM. UCNPs@ZIF-8 composites were characterized by a multi-sided prismatic structure with a large surface area and good biocompatibility. In conjunction with Molecular imprinting technology (MIT), a recognition cavity was left to capture the target. In addition, MIPs/UCNPs@ZIF-8 modified glass carbon electrodes (MIPs/UCNPs@ZIF-8/GCE) offered excellent selectivity. The modified electrode was characterized using EIS, and ECL performance was tested by CV. The results show that the intensity of ECL gradually decreases as the IM concentration increases, which proves that the presence of IM has a quenching effect on the response signal. The sensor exhibited a good linear response to IM over a wide concentration range (0.1 ng/L–1 mg/L) with a detection limit as low as 0.01 ng/L. MIPs have specific recognition and selective adsorption capabilities that improve the selective recognition and enrichment of target analytes by the sensor, thereby reducing detection time. It has been successfully applied to the determination of IM concentrations in fish, shrimp, and lettuce, providing a new means for the rapid detection of trace pesticide residues in crops and aquatic products. In addition to MIPs, the ACEK effect can also be employed to accelerate the enrichment of target analytes on the electrode surface.

Pesticides and chemical nerve agents containing organophosphorus compounds are harmful to human health. Pham et al. [51] developed a chip-based electrochemical biosensor for the detection of OP in food. The principle of the detection platform is based on the inhibition of leucovorin (DMT), a typical OP that specifically inhibits acetylcholinesterase (AChE) activity. A gold electrode modified with poly diallyl dimethyl ammonium chloride (PDDA) and oxidized nanocellulose (NC) functionalized with carbon nanotubes was used to detect OP, and the composite electrode showed a 4.8-fold higher sensitivity than the bare gold electrode. The sensor was successfully used to detect food samples spiked with organophosphates with a DMT detection limit of 4.1 nM, a quantification limit of 12.6 nM, and a linear range of 10–1000 nM. The sensor creatively uses microfluidic chip technology to miniaturize the sensor by integrating the electrodes on the chip. It has the prospect of mass production and broad application.

**Table 5:** Electrochemical biosensors for pesticide residue detection.

Analyte	Electrode	Electrochemical Method	Linearity Range	LOD	Assay Time	Ref.
PM	Au	CV	1 μM–5 μM	0.6 μM	1 min	[46]
CBZ	GCE	DPV	0.03–30 μM	9.4 nM	—	[48]
IM	GCE	CV	0.1 ng/L–1 mg/L	0.01 ng/L	—	[50]
DMT	Au	CV	10–1000 nM	4.1 nM	—	[51]

### 3.2. Heavy Metal

Heavy metal pollution is becoming a serious environmental and social problem, because the long-term accumulation of non-biodegradable heavy metal ions in the human body leads to most diseases, such as kidney failure, chronic toxic symptoms, and liver injury [52]. Therefore, lead, cadmium, chromium, and other heavy metals in food sources must be controlled to ensure public safety [53]. A typical heavy metal analysis is performed using conventional spectroscopic techniques, such as atomic absorption spectrometry (AAS) and inductively coupled plasma optical mass spectrometry (ICP-MS). However, electrochemical methods offer significant advantages over optical analytical techniques, including low cost, simplicity, and the possibility of field applications [54]. Several electrochemical biosensors for the quantitative detection of As, Hg^2+^, Cd^2+^, Pb^2+^, Cu^2+^, and Zn^2+^ were discussed below (Table 6).

Khamcharoen et al. [55] created a small electrochemical platform for detecting As (III) contamination in herbal medicines. To reduce the procedure for modification and determination, only one drop of mixed standard Au (III) and sample solution were used for electrochemical measurements using a screen-printed graphene electrode (SPGE). Square wave anode dissolution voltammetry (SWASV) was used to characterize modification and determination processes. When the reduction potential was maintained at −0.5 V, an Au–As intermetallic alloy was formed. For electrochemical determination of As (III), As was stripped away. This electrochemical sensing system can detect As (III) in the concentration range of 0.1 to 3.0 ppm with a LOD of 0.03 ppm, and a total analysis time within 3 min. The applicability and accuracy of the proposed sensor was verified by determining As (III) in the herbal sample. The advantages of simple operation, fast detection, portability, and low cost (<1 USD) make it a more powerful tool for routine monitoring and field analysis applications. It is worth noting that the proposed method is a simple and inexpensive analytical method, especially for the determination of the As (III) of herbal clocks in the context of limited resources. In situ one-step assays using standard gold solution modification SPGE is beneficial to save time, budget, and reduce the number of experimental samples.

Narouei et al. [56] reported a novel conductive nanofiber structure with a large number of nitrogen binding sites, which improved the sensitivity of SPCE for Hg^2+^ detection. The nanofibers were made of conductive copolymer polyaniline-co-o-aminophenol (PANOA), which was modified with AuNPs by electrochemical deposition. Because of the high affinity of AuNPs for Hg^2+^, the nanofibers provided high detection sensitivity for SWASV. The linear dynamic range of the sensor for Hg^2+^ is 0.8–12.0 nM, and the detection limit is 0.23 nM. The sensor is selective for Hg^2+^ under the interference of As, Pb, Cu, Zn, and Cd ions. It is also used in the detection of Hg^2+^ in river water and fish samples. This method provides a widely applicable strategy for improving the sensitivity of electrochemical sensors to heavy metal ion contamination. Compared to PANOA or AuNPs modified electrodes alone, Au-PANOA modified electrodes increase the sensitivity of the sensor to detect Hg^2+^ and have good selectivity for Hg^2+^. This strategy is very suitable for the modification of low-cost disposable SPCE electrodes, which provides a feasible solution for miniaturization and low-cost on-site detection of metal ion contamination.

**Figure 14:**
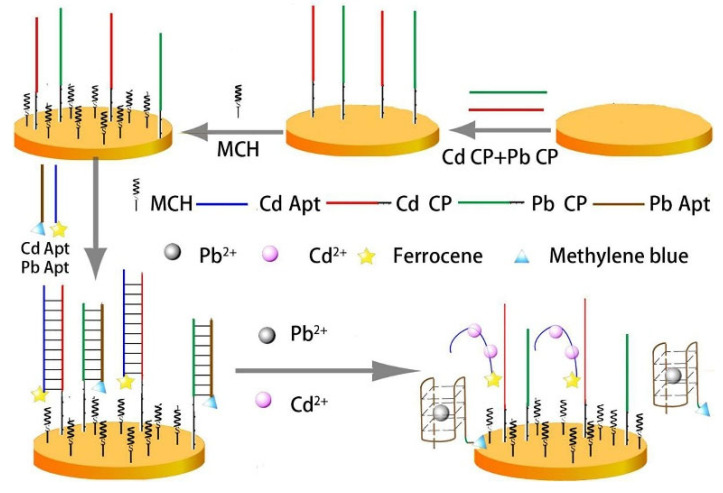
Schematic diagram of electrochemical aptamer sensor for Cd^2+^ and Pb^2+^ detection (Reprinted with permission from Ref. [57]. 2022, Yuan et al.).

Yuan et al. [57] designed an aptamer-based electrochemical aptamer sensor for the first time, which can simultaneously detect Cd^2+^ and Pb^2+^ in fruits and vegetables. The detection principle of the electrochemical aptamer sensor is shown in Figure 14. Double-stranded DNA containing aptamers is immobilized to gold electrodes by Au-S bonds. In the absence of Cd^2+^ and Pb^2+^, the sensor has a high-intensity electrochemical signal. Otherwise, after the addition of Cd^2+^ and Pb^2+^, the metal ions bind specifically to the aptamer, breaking the rigid double-stranded structure and keeping the aptamer away from the gold electrode. Therefore, the electrochemical signal of the modified electrode is weakened. The changes in electrochemical signals were measured by square wave voltammetry, and Cd^2+^ and Pb^2+^ were detected simultaneously. The results showed that the electrochemical aptamer sensor showed a linear response to Cd^2+^ and Pb^2+^ in the range of 0.1–1000 nmol/L, and the detection limits of Cd^2+^ and Pb^2+^ reached 89.31 pmol/L and 16.44 pmol/L, respectively. The electrochemical aptamase sensor showed significant response to Cd^2+^ and Pb^2+^ only. For the first time, ultra-sensitive detection of Cd^2+^ and Pb^2+^ at pmol/L levels simultaneously was achieved. Compared with other methods for simultaneous detection of Cd^2+^ and Pb^2+^, this study has the advantages of high sensitivity, strong selectivity, and simple operation.

Tan et al. [58] developed fluorinated ink/gold nanocage (FGP/AuNC) nanocomposites for simultaneous determination of heavy metals using square-wave anodic stripping voltammetry. The synergistic effect of AuNC and FGP can enhance the electrochemical active center and cation affinity, and improve the catalytic activity of FGP/AuNC composites for simultaneous electrochemical determination of Hg^2+^, Cd^2+^, Pb^2+^, Cu^2+^, and Zn^2+^. Under the conditions of buffer pH 5.0, deposition potential −1.25 V, and deposition time 140 s, this method can obtain the best results. The detection limits of FGP/AuNC electrodes were low (0.08, 0.09, 0.05, 0.19, 0.01 μg/L) and the linear ranges were wide (6–7000, 4–6000, 6–5000, 4–4000, 6–5000 μg/L). In addition, FGP/AuNC electrodes were also used for simultaneous determination of Zn^2+^, Cd^2+^, Pb^2+^, Cu^2+^, and Hg^2+^ in real samples (peanut, rapeseed and tea). The results of electrochemical method and atomic fluorescence spectrometry/inductively coupled plasma mass spectrometry are in good agreement. The method has been successfully applied to the determination of heavy metal ions in agricultural products. The sensor detects multiple heavy metal ions simultaneously, which saves the cost of the sensor and ensures the accuracy and sensitivity of the detection results. However, it has high requirements for detection samples and is susceptible to interference from impurities for the detection of complex samples, such as soil and feed.

### 3.3. Illegal Food Additives

Improper use of food additives may pose a threat to human health, so it is important to develop sensitive and selective detection methods. According to their functions, food additives can be divided into colorants, preservatives, sweeteners, thickeners, emulsifiers, promoters, and acidity regulators, etc. [59]. For the quantitative detection of food additives, many methods based on liquid chromatography, mass spectrometry and electrochemistry have been reported [60]. Compared with other methods, the electrochemical methods have the characteristics of simple operation, low cost, and high accuracy. In the following, electrochemical biosensing strategies for the detection of melamine (MEL), Sudan Red I, and clenbuterol are discussed (Table 7).

Melamine is a nitrogen-rich organic compound commonly used to increase apparent protein levels in liquid milk and milk powder. Excessive consumption of melamine can cause damage to the urinary system and increase the risk of acute kidney failure, urolithiasis, and bladder cancer in infants and children [61]. Rahman et al. [62] In order to detect melamine in an aqueous solution, the cadmium-doped antimony oxide (Cd-doped Sb_2_O_4_) nanostructures (CAO-NSs) were synthesized by low-temperature alkaline hydrothermal method. GCE was modified with CAO-NSs and its sensing performance against melamine was studied by I-V in phosphate buffer solution (PBS). Melamine undergoes a reduction reaction in the presence of CAO-NSs/GCE in PBS, and the current response gradually decreases with the increase in melamine concentration at 25.0 °C. To verify the performance of the sensor, melamine in actual milk samples was determined using CAO-NSs/GCE, and the aggregated CAO-NSs exhibited extraordinary electrocatalytic activity. CAO-NSs/GCEs have high sensitivity (3.153 μAμM^−1^cm^−2^) with a linear monitoring range of 0.05 nM–0.5 mM and a detection limit of 14.0 ± 0.05 pM. Compared with other electrochemical sensors for melamine detection, this sensor has a wider detection range and a lower detection limit at the pM level, providing better versatility. This is an effective way to develop melamine and its derivatives-sensitive sensors that can be used for safety detection in large-scale biomedical and healthcare fields.

**Figure 15:**
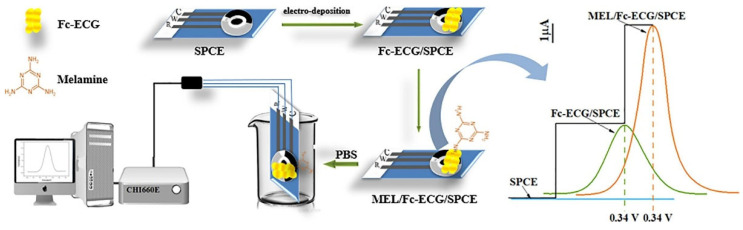
The process of making an Fc-ECG/SPCE sensor for MEL detection (Reprinted with permission from Ref. [63]. 2022, An et al.).

An et al. [63] reported a simple sensor for MEL detection in milk. Due to the low electrochemical activity of MEL, ferrocene glutathione (Fc-ECG) was used as an electron transfer medium to assist SPCE in the detection of MEL. Figure 15 depicts the preparation and signal enhancement effect of the improved SPCE, which was prepared using a step-by-step drip method for the modified electrode (MEL/Fc-ECG/SPCE). The relationship between MEL concentration and sensor current response in milk samples was investigated using DPV. The results show that since three p-π conjugated double bonds promote electron transfer, MEL significantly enhances the signal of the Fc-ECG/SPCE sensor. The sensor exhibited two linear lines in the range of 0.20–2.00 μM and 8.00–800 μM, with a detection limit of 0.13 μM. It has good stability and reproducibility, while being resistant to interference from other compounds. It has been successfully used to detect MEL in milk. However, the linear range is not continuous, and the application may be limited.

Sudan red is used in the chemical industry as a colorant in oils, fats, plastics, waxes, gasoline, shoes, printing inks, floor polish, and alcohol varnishes, and can also give red color to chili sauce, ketchup, and many other common foods [64]. However, Sudan red is a potential carcinogen, so the quantitative detection of Sudan red in food is imperative. Yang et al. [65] studied the electrochemical properties of MWCNTs/ AuNPs/ GCE for the quantitative detection of Sudan I. The surface of GCE was modified by electrodeposition of AuNPs and synthesis of MWCNTs by spray pyrolysis. The morphology and structure of the synthesized AuNPs and MWCNTs were investigated by field emission scanning electron microscopy (FESEM) and X-ray diffractometry (XRD). The results show that the MWCNTs and Au nanoparticles have high density and porous structure. The electrochemical properties of MWCNTs/AuNPs/GCE were studied by cyclic voltammetry and amperometry. Electrochemical studies showed that MWCNTs/AuNPs/GCE had high stability and sensitivity, a linear response range of 10–260 µM, and detection limit of 4 nM, which could be used for the determination of Sudan I in chili paste samples. Compared with other Sudan I electrochemical sensors, this sensor exhibits a wide linear detection range, but the detection limit is not the lowest and the sensor performance can be further improved.

Heydari et al. [66] synthesized zinc oxide nanoparticles (ZnONPs) by precipitation and used it as a novel electrocatalyst for the detection of Sudan II dye. The nanoparticles were characterized by X-ray diffraction spectroscopy and scanning electron microscopy techniques with an average diameter of 25 nm. To study the electrocatalytic effect of ZnONPs, carbon paste electrodes (CPEs) were modified with nanoparticles. The central composite rotational design and response surface method were used to optimize the influence of chemical and instrumental parameters on the oxidation reaction of Sudan II. Then, the electrochemical response of Sudan II was studied on the surface of the nanostructured modified electrode by DPV. The anode peak current of Sudan II was linearly related to its concentration in the range of 0.01–20 mM, with a detection limit of 0.0017 mM. The application of ZnONPs increased the active area of the electrode and improved the electron transfer between Sudan Red II and the electrode surface, resulting in a 2.4 folds increase in the voltammetric peak current of this sensor over the bare electrode. In addition, the method was satisfactory for the detection of Sudan II in chili sauce. The detection limit of this nanostructured sensor was superior compared to other electrochemical sensors for the detection of Sudan II.

Clenbuterol (CLB) was originally used to treat human depression and lung disease [67]. In recent years, clenbuterol has been illegally used to feed live pigs because of its anabolic effects that promote muscle growth and reduce fat mass [68]. However, it can remain in the muscle tissue and internal organs of animals that are being raised, endangering the health of those who eat it. Sun et al. [68] prepared two kinds of rGO/Fe_3_O_4_ nanocomposites by solvothermal and hydrothermal methods and modified them onto the surface of glassy carbon electrodes to prepare a Clenbuterol electrochemical sensor for direct electrochemical detection of clenbuterol residues in animal metabolism. CV and DPV were used for electrochemical characterization and measurement, and the detection performance of the sensor for clenbuterol was investigated. The electrochemical sensor can detect clenbuterol linearly in the range of 1–128 μm, and the detection limit is 120 nM (S/N = 3). The electrochemical sensor has been successfully used to detect clenbuterol in pig urine. Compared to other clenbuterol sensors, this sensor does not have an outstanding detection limit, but has a wider linear range and is more versatile.

Jing et al. [69] proposed a new method for electrochemical detection of clenbuterol using boron carbon-oxynitrogen (BCNO) nanosheets as a sensing medium. BCNO nanosheets were prepared using a sodium chloride/potassium chloride eutectic mixture as a molten base, and the samples prepared were BCNO crystal with a nano-sheet structure. Modification of BCNO on GCE can significantly improve electron transport capacity and provide more electrochemically active sites. Clenbuterol was detected by using differential pulse dissolution voltammetry (DPSV), and the DPSV signal increases as the CLB increases. BCNO-modified GCE (BCNO/GCE) exhibited superior analytical performance when detecting Clenbuterol. The sensor had a lower detection limit of 17 nM and a linear concentration range of 0.03–16.0 μM. Finally, the method was applied to the determination of clenbuterol in porcine serum and tablets, and satisfactory recovery was obtained. The results suggest that BCNO nanostructures are expected to be candidates for electrochemical sensors. The sensor has good interference immunity, repeatability and stability for CLB detection. It shows a wide linear range and low LOD compared to the analytical performance of other electrochemical sensors, which is superior to most previous reports.

**Table 7:** Electrochemical biosensors for the detection of illicit additives.

Analyte	Electrode	Electrochemical Method	Linearity Range	LOD	Assay Time	Ref.
Melamine	GCE	I-V	0.05 nM–0.5 mM	14.0 ± 0.05 pM	—	[62]
	SPCE	DPV	0.20–2.00 μΜ 8.00–800 μM	0.13 μM	—	[63]
Sudan red	GCE	CV Amperometry	10–260 µM	4 nM	—	[65]
	CPE	DPV	0.01–20 mM	0.0017 mM	—	[66]
Clenbuterol	GCE	CV DPV	1–128 μM	120 nM	—	[68]
	GCE	DPSV	0.03–16.0 μM	17 nM	—	[69]

## 4. Genetically Modified Crops

Genetically Modified Crops (GMC) are plants in which genes with target traits are modified by genetic engineering techniques and then introduced into the genome of the recipient plant [70]. These exogenous genes are not only stably inherited in the offspring, but can also lead to beneficial traits such as insect resistance, herbicide resistance, and disease resistance in the crop. However, the biosafety of GMC has been controversial. Currently, the detection of GMC components mainly includes gene nucleic acid detection, protein detection, and metabolite detection [70]. although traditional detection methods such as PCR, ELISA, and HPLC are mature and reliable, they cannot meet the practical needs of high speed and low cost. Therefore, a fast, accurate, and low-cost field detection platform for transgenic crops is needed. The following is a discussion of electrochemical biosensors for GM soybean and maize detection (Table 8).

**Figure 16:**
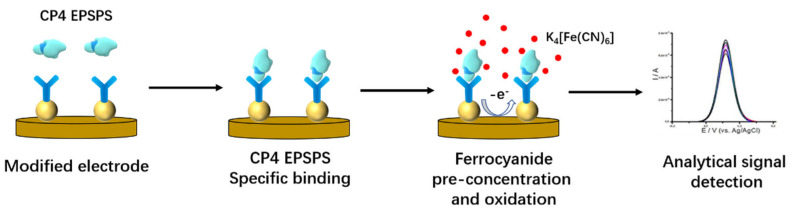
Schematic diagram of the electrochemical immunosensor for the detection of CP4 EPSPS (Adapted with permission from Ref. [71]. 2022, Marcos et al.).

Marcos et al. [71] developed an electrochemical immunosensor for the detection of the transgenic soy protein CP4 EPSPS in soybean seeds, which does not require a labeling and signal amplification system. The sensor schematic is shown in Figure 16. The CP4 EPSPS antibodies were first modified on the gold electrodes, and the modified gold electrode was incubated in soy protein solutions of different concentrations at 37 °C for 30 min. After washing with ultrapure water, the electrodes were immersed in an electrochemical cell containing K_4_[Fe(CN)_6_] (1 mM) and LiCl (0.1 M) solutions. Electrochemical measurements were performed using SWV, and the peak current was linearly related to the concentration of soy protein in the range of 0.005–0.3 mg/mL, with a detection limit of 38 ng/mL CP4 EPSPS (below 0.00038% CP4 EPSPS). Since many countries recommend labeling foods containing higher than 0.9% CP4 EPSPS, the detection limit of this sensor meets the detection needs.

Gao et al. [72] also established a label-free electrochemical sensing platform for the detection of transgenic soybean SHZD32-1. Soybean SHZD32-1 seeds were ground into powder and then genomic DNA samples were extracted by a CTAB-based method. GCDs modified on the SPCE surface can be attached to single-stranded DNA probes via Au-S bonds while improving the conductivity of the DNA sensor. After binding of the DNA probes to transgenic soybean DNA, the electron transfer resistance (R_et_) on the sensor surface was quantified by the R_et_ response increased with logarithmic increase in target DNA concentration over a linear range of 1.0 × 10^−7^–1.0 × 10^−13^ M, with a detection limit of 3.1 × 10^−14^ M. This label-free sensor is made by inserting the SPCE into a handheld EI analyzer is conveniently fabricated, demonstrating simplicity of construction and operation, requiring no additional indicators or cumbersome procedures, and can be used in a friendly manner by non-specialists.

**Figure 17:**
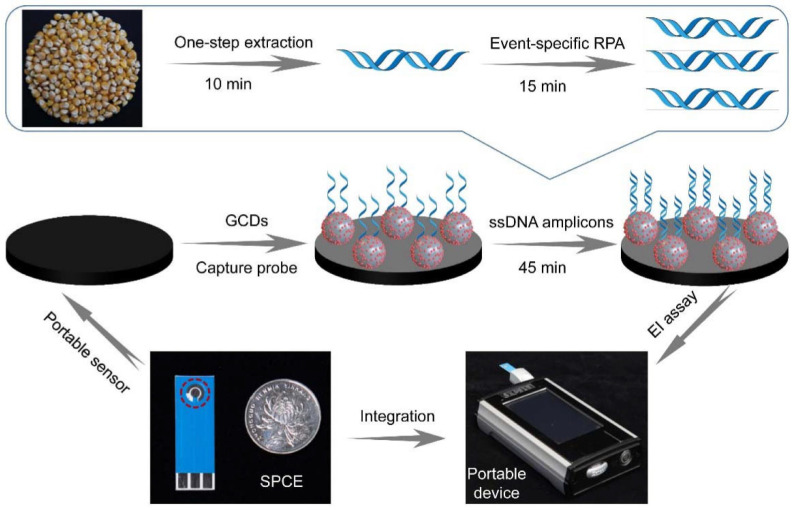
Fabrication process of EI gene sensor based on GCD (Reprinted with permission from Ref. [73]. 2022, Cui et al.).

Cui et al. [73] developed a label-free electrochemical impedance gene sensor using gold carbon dots (GCDs) and an easy-to-use portable device. It consists of a handheld electrochemical impedance (EI) analyzer equipped with a coin-sized SPCE. Figure 17 shows the preparation process of this sensor, GCDs were used to modify a screen-printed carbon electrode and capture probes were immobilized by Au-S bonding. Transgenic maize sample DNA was extracted using a one-step extraction method with direct plant lysis buffer and amplified by recombinase polymerase amplification (RPA). The capture probes immobilized on the sensor were identical to the forward RPA primer. After the amplification products bound to the capture probes, the EI signal increased due to the formation of a biocomplex that hindered the interfacial electron transfer. The proposed genetic sensor combined with RPA can detect maize Ruifeng12-5 in a linear range of 0.10–5.0% with a detection limit of 0.10%, roughly calculated as 36 copies/µL based on the size of the maize haploid genome. The sensor device is simple to prepare and does not require expensive instruments or specialized personnel and has wide application prospects.

## 5. Summary and Outlook

As food imports and consumption continue to grow in all regions of the world, there is an increasing need for rapid and accurate testing of potentially contaminated foods. Electrochemical biosensors offer a platform to address this challenge due to their rapid testing, portability, and low cost. This article provides a detailed review of electrochemical biosensors for the detection of biological food contaminants, chemical food contaminants, and genetically modified crops.

Although these sensors have shown excellent performance, further research is needed. Not only do researchers need to simplify sample preparation, but also need to enable sensors to detect target analytes in samples at very low concentrations. Research shows that the inclusion of nanomaterials on the electrode surface increases the conductivity and surface area of the electrode, loading more antibodies and markers and improving the electrocatalytic performance of the sensor. In another case, adding nitrogen binding sites or doping nitrogen on the electrode surface can improve the electrical signal and improve the sensitivity of the sensor. Furthermore, real-time monitoring needs to be done on-site. In the future, with the emergence of new nanomaterials, microfluidics, and molecular biology techniques, more new electrochemical biosensors will be designed with higher detection performance to ensure food safety.

## Figures and Tables

**Table 4:** Electrochemical biosensors for soy allergen detection.

Analyte	Electrode	Electrochemical Method	Linearity Range	LOD	Assay Time	Ref.
Genistein	Carbon	DPV	100 ppb–10 ppm	100 ppb	—	[41]
Ara h 1 Ara h 6	SPCE	LSV	0–1000 ng/mL 0–1.0 ng/mL	5.2 ng/mL 0.017 ng/mL	2 h 20 min	[42]

**Table 6:** Electrochemical biosensors for heavy metal detection.

Analyte	Electrode	Electrochemical Method	Linearity Range	LOD	Assay Time	Ref.
As	SPGE	SWASV	0.1–3.0 ppm	0.03 ppm	<3 min	[55]
Hg^2+^	SPCE	SWASV	0.8–12.0 nM	0.23 nM	—	[56]
Cd^2+^ Pb^2+^	Au	SWV	0.1–1000 nmol/L	89.31 pmol/L 16.44 pmol/L	15 min	[57]
Hg^2+^ Cd^2+^ Pb^2+^ Cu^2+^ Zn^2+^	GCE	SWASV	6–7000, 4–6000, 6–5000, 4–4000, 6–5000 μg/L	0.08, 0.09, 0.05, 0.19, 0.01 μg/L	—	[58]

**Table 8:** Electrochemical biosensors for the detection of GMC.

Analyte	Electrode	Electrochemical Method	Linearity Range	LOD	Assay Time	Ref.
CP4 EPSPS	Au	SWV	0.005–0.3 mg/mL	38 ng/mL	—	[71]
SHZD32-1	SPCE	EIS	1.0 × 10^−7^–1.0 × 10^−13^ M	3.1 × 10^−14^ M	—	[72]
Ruifeng12-5	SPCE	EIS	0.10–5.0%	0.10%	—	[73]

## Data Availability

Not applicable.
